# Exploring sexual function in adrenal insufficiency: findings from the Dual RElease hydrocortisone versus conventionAl glucocorticoid replaceMent therapy in hypocortisolism (DREAM) trial

**DOI:** 10.1111/andr.13635

**Published:** 2024-03-28

**Authors:** Valeria Hasenmajer, Dario De Alcubierre, Davide Ferrari, Marianna Minnetti, Ilaria Bonaventura, Riccardo Pofi, Chiara Simeoli, Alessandra Tomaselli, Francesca Sciarra, Grazia Bottillo, Francesco Angelini, Alessia Cozzolino, Mary Anna Venneri, Emmanuele A. Jannini, Daniele Gianfrilli, Rosario Pivonello, Andrea M. Isidori

**Affiliations:** ^1^ Department of Experimental Medicine “Sapienza” University of Rome Rome Italy; ^2^ Inserm U1052, CNRS UMR5286 Cancer Research Center of Lyon Claude Bernard Lyon 1 University Lyon France; ^3^ Oxford Centre for Diabetes Endocrinology and Metabolism NIHR Oxford Biomedical Research Centre Churchill Hospital University of Oxford Oxford UK; ^4^ Dipartimento di Medicina Clinica e Chirurgia Sezione di Endocrinologia, Università Federico II di Napoli Naples Italy; ^5^ Laboratory of Cutaneous Physiopathology and Integrated Centre for Metabolomics Research San Gallicano Dermatological Institute ‐ IRCCS Rome Italy; ^6^ Department of Systems Medicine Endocrinology and Medical Sexology (ENDOSEX) University of Rome Tor Vergata Rome Italy; ^7^ Centre for Rare Diseases (Endo‐ERN accredited) Policlinico Umberto I Rome Italy

**Keywords:** adrenal insufficiency, dual‐release hydrocortisone, erectile dysfunction, sexual function

## Abstract

**Background:**

Data on sexual function in patients with adrenal insufficiency are scarce and largely controversial.

**Objectives:**

To investigate sexual dysfunction in patients with primary and secondary adrenal insufficiency and the effects of switching to once‐daily dual‐release hydrocortisone on sexual function in outcome assessors blinded, randomized, multicenter, active comparator clinical trial.

**Materials and methods:**

Eighty‐nine adrenal insufficiency patients on conventional, multiple daily doses of glucocorticoid replacement, enrolled in the Dual RElease hydrocortisone versus conventionAl glucocorticoid replaceMent in hypocortisolism (DREAM) trial, were randomly assigned to continue their therapy or to switch to an equivalent dose of dual‐release hydrocortisone. Sixty‐three patients (34 women) consented to sex steroid measurements and questionnaires completion for quality of life (Addison's disease‐specific quality‐of‐life questionnaire) and sexual function evaluation (female sexual function index for women, International Index of Erectile Function‐Erectile Function for men) at baseline and 24 weeks after randomization.

**Results:**

At baseline, sexual dysfunction was observed in 41% of women and 59% of men with adrenal insufficiency. In both sexes, no associations were found between sexual function and hormone levels, whereas Addison's disease‐specific quality‐of‐life questionnaire total and fatigue domain scores positively correlated with total female sexual function index and International Index of Erectile Function‐Erectile Function scores. At 24 weeks, there was no significant difference either in sexual function or sex steroid levels between study groups. In the dual‐release hydrocortisone group, the variation in the female sexual function index desire domain score was positively associated with the change in Addison's disease‐specific quality‐of‐life questionnaire's symptom domain score (*ρ* = 0.478, *p* = 0.045).

**Discussion:**

Sexual dysfunction is common in adrenal insufficiency patients and is likely explained by multiple factors. dual‐release hydrocortisone treatment is not directly associated with sexual function improvement, but an indirect effect mediated by quality‐of‐life amelioration cannot be excluded.

## 1 INTRODUCTION

Adrenal insufficiency (AI) is characterized by inadequate hormonal secretion by the adrenal cortex, which can occur either due to a primary adrenal gland impairment (PAI) or to a secondary deficiency of adrenocorticotropic hormone (ACTH) secretion at the pituitary level (SAI).^1^, ^2^

AI is associated with higher mortality rates^3^, ^4^ and several systemic comorbidities, including metabolic, immune, and bone‐related derangements,^5^, ^6^, ^7^, ^8^, ^9^, ^10^ that are all known to impact sexual function in the general population.^11^, ^12^, ^13^ Nevertheless, in these patients, this remains an underexplored and unaddressed aspect, despite the fact that several disease‐specific factors could also affect sexual health, including impaired gonadal function (i.e., due to concurrent premature ovarian failure in PAI or hypogonadotropic hypogonadism in SAI), increased fatigue, decreased vitality and quality of life (QoL), and higher cardiovascular risk.^5^, ^14^, ^15^, ^16^, ^17^

In addition, individuals with PAI generally have abnormally low levels of adrenal androgens that may further impact libido and sexual function, especially in women following gonadal failure, either due to physiological menopause or as a result of premature primary ovarian insufficiency.^2^, ^18^, ^19^

To date, few studies have focused on sexuality in females with AI, based on the idea that the lack of adrenal androgen secretion could affect this aspect.^20^, ^21^ Despite the convincing premises, the actual impact of adrenal androgen deficiency has been questioned, as sexual function has been found not to be significantly improved by dehydroepiandrosterone (DHEA) replacement either in women with AI^22^ or untreated estrogen deficiency.^23^

Data on the prevalence of sexual dysfunction in male patients with AI are even more scarce. So far, only one study has assessed the prevalence of erectile dysfunction (ED) in newly diagnosed AI before and after two months of treatment^24^; however, data about sexuality in male patients with PAI on chronic glucocorticoid (GC) and mineralocorticoid replacement therapy have never been obtained.

Dual‐release hydrocortisone (DR‐HC), a novel formulation mimicking the physiological circadian rhythm of cortisol secretion, has been associated with an improvement in QoL and metabolic parameters in AI patients^8^, ^25^, ^26^; moreover, lifestyle and metabolic interventions have been reportedly associated with an improvement in sexual dysfunction in the general population.^11^, ^27^ Nevertheless, the impact of a more physiological treatment on sexual function in patients with AI has not been fully explored yet.

In the context of the Dual RElease hydrocortisone versus conventionAl glucocorticoid replaceMent in hypocortisolism (DREAM) trial,^8^ a randomized‐controlled study on DR‐HC in PAI and SAI patients, we aimed to conduct a comprehensive assessment of sexual function in men and women with AI on long‐term GC therapy before and after the switch to DR‐HC, to investigate whether a more physiological replacement could provide a beneficial effect on sexuality.

## 2 MATERIALS AND METHODS

### 2.1 Study design and participants

The DREAM study was a multicenter, randomized, two‐armed, outcome assessor‐blinded (independent), active comparator, controlled clinical trial. In the original study, 89 AI patients were randomly assigned to either continue their multiple daily doses of conventional glucocorticoid treatment (CT group) or to switch to an equivalent dose of once‐daily DR‐HC (DR‐HC group). All patients were evaluated at baseline and after 24 weeks. The daily dose of GC remained constant throughout the study timeframe. The primary outcome was body weight change, while secondary outcomes included changes in immune cell phenotype, susceptibility to infections, metabolic profile, and QoL, including sexual function.

All patients provided written informed consent and the trial was approved by the local review board at Sapienza University, conducted in accordance with the Declaration of Helsinki, and performed between March 2014 and June 2016 (ClinicalTrials.gov identifier NCT02277587) in two Italian referral centers for adrenal and pituitary disorders (Sapienza University of Rome and Federico II University of Naples).

**FIGURE 1:**
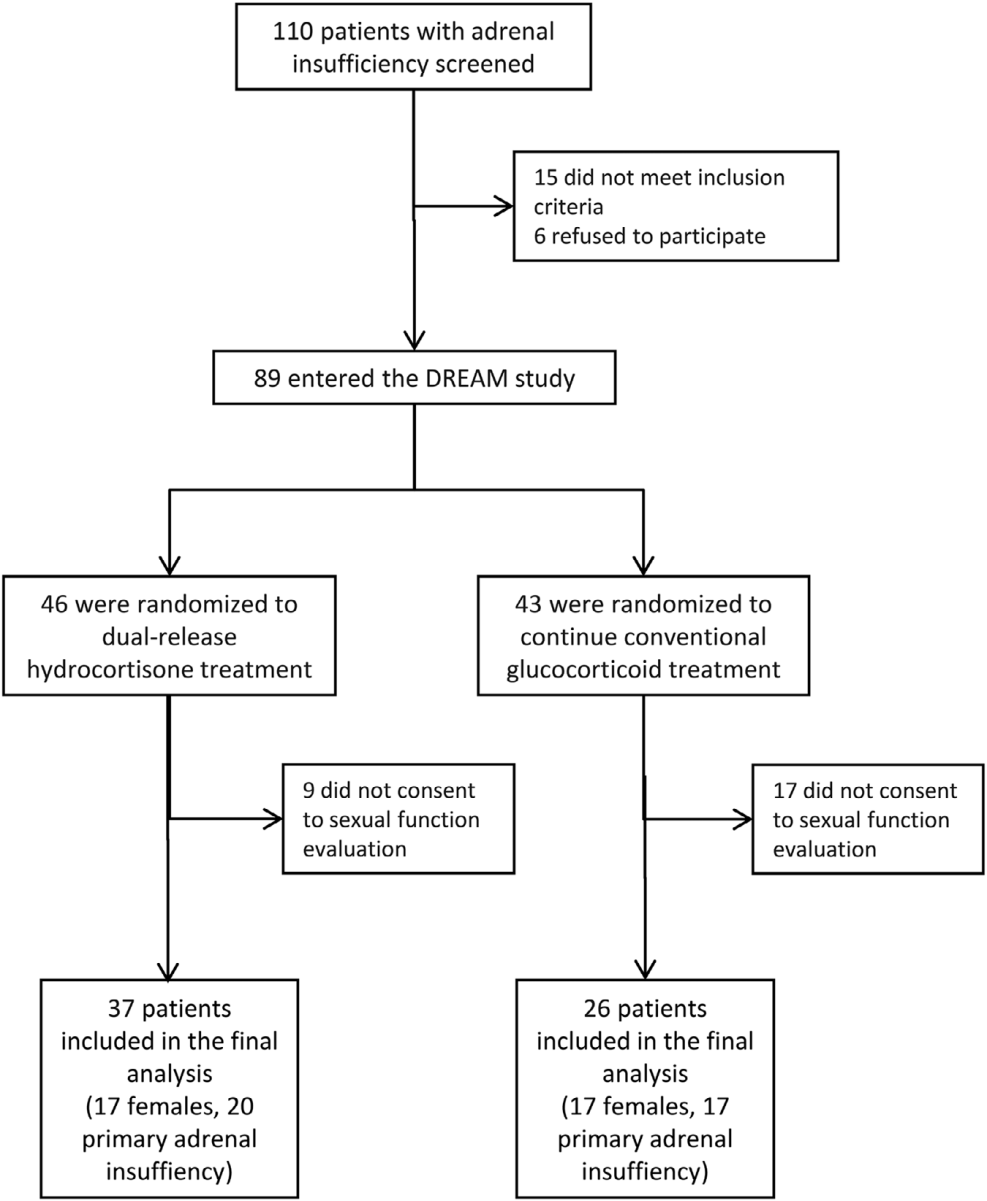
Study flowchart. Flowchart detailing the patient selection process.

For the current analyses, AI patients were asked to provide informed consent for sexual function evaluation and sex steroids analysis. The patient selection process is detailed in Figure 1.

Of the 89 patients who participated in the original study, 63 consented to sexual function analysis through questionnaire completion and hormonal evaluation. Data regarding medical history and anthropometric parameters were collected, as previously reported in the core study.^8^ Hormone deficiencies were adequately replaced starting at least 6 months prior to study enrolment and throughout the study duration.

### 2.2 Study procedures

#### 2.2.1 Sexual function questionnaires

Patients were asked to complete the Female Sexual Function Inventory (FSFI) or the 6‐item International Index of Erectile Function‐Erectile Function (IIEF‐EF) questionnaires at baseline and after 24 weeks.

For the FSFI questionnaire, according to previous studies evaluating cut‐off scores,^28^, ^29^ we considered our patients to have sexual dysfunction if the total score was ≤26.55 after adding individual domain scores for pain, satisfaction, orgasm, lubrication, arousal, and desire.

Both prevalence and severity of ED were evaluated via the IIEF in its shorter, 6‐item version, which only assesses the erectile domain of the sexual function (IIEF‐EF); in accordance with published criteria, scores ≥26 identified patients with no ED.^30^

#### 2.2.2 Sex hormones evaluation

At baseline and after 24 weeks, patients underwent blood sampling for hormone evaluation. Blood draws were performed in the morning between 8:00 and 9:00, following an overnight fast and two hours after the regular morning GC dose was taken. For pre‐menopausal female patients, blood sampling was performed between the 3rd and the 5th day of the menstrual cycle. Hormone assays for DHEAS, 17‐OH‐Progesterone, Testosterone, Estradiol, and Androstenedione, were carried out at “Sapienza” University with high‐performance liquid chromatography‐mass spectrometry. Detailed methods of steroidomic analysis have been described elsewhere.^31^

#### 2.2.3 QoL assessment

QoL was assessed via Addison's disease‐specific QoL questionnaire (AddiQoL), which comprises 30 items categorized into four domains (fatigue, emotion, symptoms, and miscellaneous). Scores were calculated in line with published data, with higher scores reflecting a better QoL.^32^ Data regarding QoL‐related changes following DR‐HC treatment were previously published in the core study.^8^

### 2.3 Statistical analysis

The statistical plan of the entire study has been previously reported,^8^ and the full pre‐specified plan is available online (https://web.uniroma1.it/dip_dms/ricerca/trials‐clinici). The normality of distribution was assessed by the Shapiro‐Wilk test. Correlations between sexual function questionnaires and clinical and biochemical variables were estimated by Pearson's correlation coefficient or Spearman's rank correlation coefficient, according to the distribution of variables. Student's t‐test or the Mann‐Whitney U test was performed to compare continuous variables between groups. Differences between categorical variables were evaluated via χ^2^ statistics. The estimated means with a 95% confidence interval of treatment differences (ETD) in change from baseline to week 24 were analyzed with an ANCOVA model that included treatment as a fixed effect and baseline outcome, body mass index (BMI), age, type and duration of AI, and diabetes mellitus as covariates. Bonferroni correction for multiple comparisons was applied.

## 3 RESULTS

Overall, 63 of 89 patients (71%, 34 women and 29 males) completed the questionnaires investigating sexual function at baseline. The dropout rate for questionnaire completion at 24 weeks was 6% for females and 13% for males. Patients not consenting to sexual function evaluation were older (median age: 55 [48–61] vs. 45 [42–49] years, *p* = 0.012) and had longer disease duration (102 [36–207] vs. 36 [12–96] months, *p* = 0.023) compared to those who filled the questionnaire; the remaining main clinical and demographic parameters, including AI etiology (*p* = 0.203), baseline GC daily dose (*p* = 0.947), sex (*p* = 0.210), diabetes (*p* = 0.375), and menopause prevalence (*p* = 0.575) were otherwise similar between the two groups.

Baseline characteristics did not differ between patients allocated to switch to once‐daily DR‐HC and those randomized to continue CT (Table 1). In the whole cohort, eight patients (12.7%, four males, two PAI) presented with diabetes mellitus, which was well‐controlled (HbA1c < 7%) throughout the study duration in all cases. Fourteen women (41.1%, 8 PAI) were menopausal, and nine males (31%, all SAI) were on stable testosterone replacement for hypogonadism. No patient was receiving DHEA or estrogen replacement therapy, nor hormonal contraceptives or phosphodiesterase 5 inhibitors during the study timeframe.

**TABLE 1:** Baseline characteristics of the entire cohort completing sexual function questionnaires.

	Whole cohort *N* = 63	Randomized to switch treatment *N* = 38	Randomized to standard treatment *N* = 25	*p*
Age (years)	45.6 ± 14.0	45.0 ± 14.0	46.5 ± 14.2	0.680
Sex (F/M)	34/29	17/21	17/8	0.070
**Clinical features**				
Primary/Secondary AI	37/26	21/17	16/9	0.491
AI duration (months)	36.0 (12–96)	46.5 (24–108)	20.0 (12–72)	0.075
Menopause	14 (41.1%)	6 (35.2%)	8 (47%)	0.486
BMI (kg/m^2^)	24.2 (21–28)	26.0 (21–29)	22.7 (20–26)	0.084
**Comorbidities**				
Diabetes mellitus	8 (12.7%)	6 (15.8%)	2 (8%)	0.364
Other autoimmune disorders	21 (33.3%)	12 (31.6%)	9 (36%)	0.771
Pituitary tumor/surgery	21 (33.3%)	15 (39.4%)	6 (24%)	0.202
Hypogonadism^*^	9 (31% of males)	8 (38.1%)	1 (11.1%)	0.139
Adrenalectomy	3 (4.7%)	2 (5.3%)	1 (4%)	0.818
**GC replacement**				
Hydrocortisone	33 (52.4%)	22 (57.8%)	11 (44%)	0.280
BID/TID^§^	29/4	20/2	9/2	
Cortisone acetate	30 (47.6%)	16 (42.2%)	14 (56%)	
BID/TID^§^	30/0	16/0	14/0	
Daily HC equivalent dose (mg/m^2^/24 h)	13.2 (11–18)	12.6 (11–17)	14.9 (12–24)	0.067

Continuous variables are expressed as mean ± SD or median (interquartile range), as appropriate. Categorical variables are expressed as count (*n*) and percentages (%).

Abbreviations: AI, adrenal insufficiency; BID, two times a day; BMI, body mass index; F, female; GC, glucocorticoid; HC, hydrocortisone; M, male; TID, three times a day.

* All patients presenting with hypogonadism were on adequate, stable testosterone replacement therapy throughout the study duration.

Of note, 22 (64.7%) of the 34 female patients completing the questionnaires were sexually active; following randomization, they were evenly allocated in the two study groups (11 women for each treatment group).

Moreover, out of the 29 male patients included in the sexual function analysis, 20 (69%) were allocated to the treatment switch group, whereas nine (31%) were randomized to continue their previous treatment.

### 3.1 Female population

#### 3.1.1 Baseline assessment

Data regarding baseline hormone levels and sexual function of the female cohort with AI are shown in Table 2. Among the 22 sexually active women, nine (40.9%) showed FSFI scores indicative of sexual dysfunction at baseline. Within the entire cohort, including both sexually active and inactive women, 94% exhibited diminished sexual desire and this reduction was observed in all post‐menopausal patients.

**TABLE 2:** Baseline hormonal status and questionnaire scores of female patients, stratified according to gonadal status and etiology of adrenal insufficiency.

	Pre‐menopause *N* = 20	Post‐menopause *N* = 14	*p*	PAI *N* = 25	SAI *N* = 9	*p*
Age (years)	40.4 ± 8.7	51.2 ± 0.9	<0.001	40.0 ± 8.8	48.8 ± 5.8	0.035
**Sex steroids evaluation**						
Estradiol (pg/ml)	29.0 (5.2)	16.7 (4.6)	0.073	20.9 (4.2)	24.8 (7.6)	0.607
Testosterone (ng/mL)	0.14 (0.04)	0.04 (0.01)	0.051	0.08 (0.03)	0.07 (0.02)	0.745
Androstenedione (ng/mL)	1.60 (0.66)	0.38 (0.10)	0.090	0.82 (0.37)	0.57 (0.64)	0.386
DHEAS (ng/mL)	131.8 (81.6)	43.2 (12.)	0.777	55.0 (20.8)	86.6 (54.4)	0.897
17‐OH‐Progesterone (ng/mL)	0.54 (0.08)	0.66 (0.08)	0.270	0.62 (0.07)	0.62 (0.13)	0.951
**FSFI questionnaire**						
Desire	4.0 ± 1.4	2.7 ± 1.6	0.166	4.1 ± 1.4	3.0 ± 1.5	0.203
Arousal^a^	4.5 ± 1.2	2.6 ± 2.6	0.081	4.6 ± 1.1	3.1 ± 2.4	0.294
Orgasm^a^	4.5 ± 1.3	3.2 ± 2.3	0.262	4.6 ± 1.3	3.4 ± 1.9	0.115
Lubrication^a^	3.9 ± 1.7	3.2 ± 2.4	0.712	3.9 ± 1.8	3.5 ± 1.9	0.802
Satisfaction^a^	4.6 ± 1.6	3.1 ± 2.2	0.118	4.6 ± 1.5	3.7 ± 2.3	0.367
Pain^a^	4.6 ± 2.1	3.3 ± 2.5	0.300	4.5 ± 2.2	4.2 ± 2.4	0.914
FSFI total score^a^	26.4 ± 7.6	18.1 ± 13.3	0.118	26.3 ± 7.7	20.9 ± 12.0	0.231
Female sexual dysfunction	7 (38.8%)	2 (50%)	0.683	6 (37.5%)	3 (50%)	0.595

Continuous variables are expressed as mean (SEM) or mean ± SD, as appropriate. Categorical variables are expressed as count (*n*) and percentages (%).

Abbreviations: DHEAS, dehydroepiandrosterone sulfate; FSFI, female sexual function index; PAI, primary adrenal insufficiency; SAI, secondary adrenal insufficiency.

^a^ Score relative to this domain was only investigated in sexually active women.

In sexually active women, no associations were observed between sexual function and sex steroid levels, except for a trend toward a positive association between the FSFI lubrication domain score and testosterone levels (*ρ* = 0.610, *p* = 0.081). Furthermore, there were no significant associations between the desire domain score and sex steroid levels when considering the whole female cohort.

No correlations were detected between any of the sexual function domains and age, disease duration, and daily body surface area‐adjusted GC dose at baseline.

Conversely, the overall AddiQoL score and the fatigue AddiQoL domain score positively correlated with the desire (*ρ* = 0.566, *p* = 0.014; *ρ* = 0.499, *p* = 0.035), arousal (*ρ* = 0.639, *p* = 0.004; *ρ* = 0.472, *p* = 0.048), satisfaction (*ρ* = 0.602, *p* = 0.008; *ρ* = 0.554, *p* = 0.017) and pain (*ρ* = 0.499, *p* = 0.035; *ρ* = 0.459, *p* = 0.050) domain scores, as well as with total FSFI total score (*ρ* = 0.549, *p* = 0.018; *ρ* = 0.487, *p* = 0.040). Lastly, the satisfaction domain was positively associated with the AddiQoL symptom domain score (higher scores indicating fewer symptoms) (*ρ* = 0.478, *p* = 0.045).

Interestingly, subgroup analysis showed similar rates of sexual dysfunction between sexually active menopausal and premenopausal patients (50% vs. 38.8%, *p* = 0.683) (Table 2), even though the prevalence of sexual activity was significantly lower in menopausal women (90% vs. 28.5%, *p* < 0.001). No significant correlations between hormone levels and questionnaire scores were detected by subgroup analyses on pre‐menopausal and menopausal sexually active women.

Furthermore, the baseline rate of overall sexual dysfunction and questionnaire scores was not different between PAI and SAI (37.5% vs. 50%, *p* = 0.595) (Table 2), and patients randomized to DR‐HC and CT had similar scores for total and single‐domain FSFI scores and prevalence of sexual dysfunction (45.4% vs. 36.3%, *p* = 0.665).

#### 3.1.2 Week 24

**FIGURE 2:**
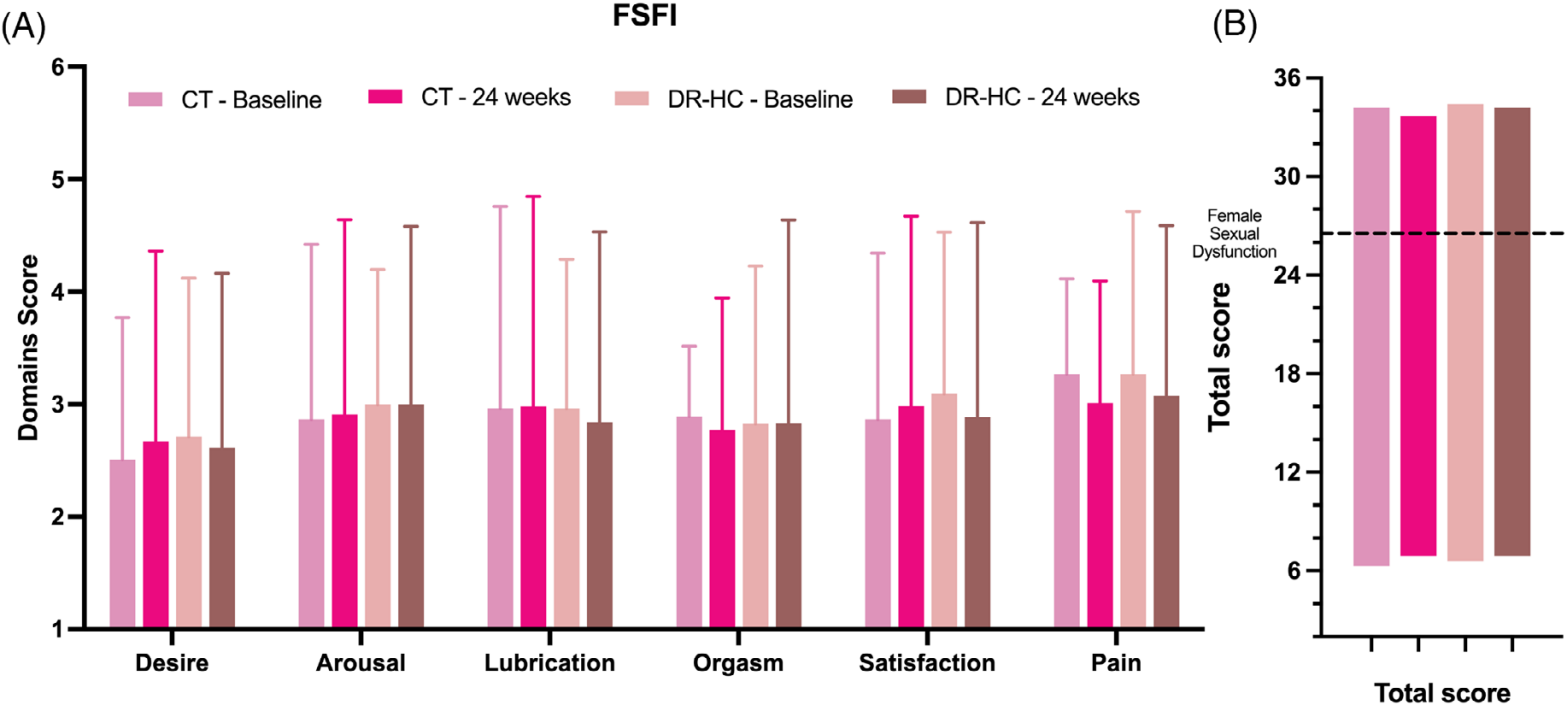
Longitudinal evaluation of female sexual function index (FSFI) scores. FSFI domains (A) and total (B) scores of the two treatment groups at baseline and after 24 weeks from randomization. Data are reported as mean (SD). CT = Conventional therapy group, DR‐HC = Dual Release Hydrocortisone group.

After 24 weeks, sexual dysfunction was observed in 10 women (45.4%), with a mean FSFI total score of 24.6 (9.0) in sexually active women. Compared to baseline, no significant changes were detected in either total or single‐domain FSFI scores in sexually active women in both treatment groups (Figure 2).

Moreover, the variations in FSFI scores did not significantly differ between the DRHC and CT groups, even after adjusting for age, BMI, type of AI, menopause, baseline FSFI score, presence of diabetes mellitus or disease duration [mean estimated difference: −2.8 (−11.4–5.9); *p* = 0.493)].

No significant changes in sex steroid levels were observed compared to baseline following the switch to DR‐HC (Table S1) and no associations between hormonal levels and sexual function questionnaire scores were observed.

Lastly, when considering both sexually active and inactive women, the DR‐HC group showed a positive association between the variation in the FSFI desire score and the change in the AddiQoL's symptom domain score (*ρ* = 0.587, *p* = 0.045).

### 3.2 Male population

#### 3.2.1 Baseline assessment

The main findings concerning baseline hormonal levels and erectile function in the male cohort are summarized in Table 3.

**TABLE 3:** Baseline hormonal status and questionnaire scores of male patients.

	Whole cohort *N* = 29	PAI *N* = 12	SAI *N* = 17	*p*
Age (years)	46.2 ± 17.3	40.7 ± 15.4	50.0 ± 18.0	0.160
**Sex steroids evaluation**				
Estradiol (pg/ml)	27.1 (5.2)	19.5 (12.5)	29.0 (5.8)	0.296
Testosterone (ng/mL)	3.34 (0.74)	3.91 (2.51)	3.23 (0.81)	0.878
Androstenedione (ng/mL)	1.04 (0.26)	1.57 (1.39)	0.93 (0.22)	0.830
DHEAS (ng/mL)	135.0 (55.0)	84.9 (43.5)	104.32 (54.6)	0.602
17‐OH‐Progesterone (ng/mL)	0.56 (0.10)	0.69 (0.06)	0.53 (0.12)	0.558
**Erectile function**				
IIEF‐EF score (0–30)	19.3 ± 10.0	23.4 ± 7.4	16.4 ± 10.8	0.048
Erectile dysfunction, *n* (%)	17 (58.6%)	6 (50%)	11 (64.7%)	0.428

Continuous variables are expressed as mean (SEM) or mean ± SD, as appropriate. Categorical variables are expressed as count (*n*) and percentages (%).

Abbreviations: DHEAS, dehydroepiandrosterone sulfate; IIEF‐EF = International Index of Erectile Function‐Erectile Function; PAI, primary adrenal insufficiency; SAI, secondary adrenal insufficiency.

In the whole cohort, the presence of ED was observed in 17 patients (58.6%). A subsequent stratification according to age revealed an overall progressive worsening of sexual function with age: the prevalence of ED was 30% in patients aged 20–39, 55% in patients aged 40–59, and 90% in patients aged 60–79. No significant difference in the prevalence of sexual dysfunction was observed compared to female patients (58.6% vs. 40.9%, *Χ* ^2 ^= .065, *p* = 0.799).

**FIGURE 3:**
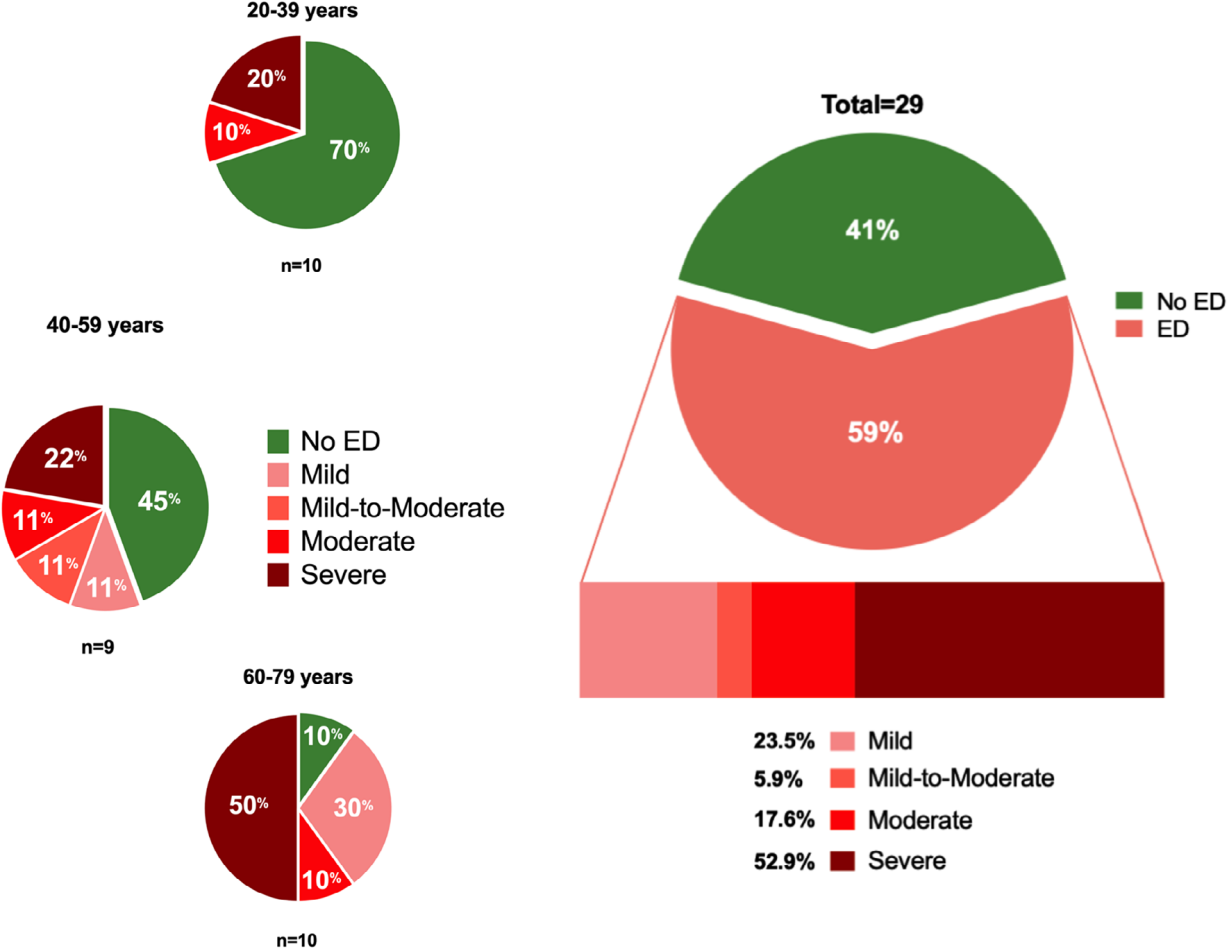
Global and age‐stratified prevalence and severity of erectile dysfunction (ED) in male patients. Percentages describing the overall prevalence of ED in patients with adrenal insufficiency, as well as its distribution and severity across different age groups.

The severity of ED also showed a different pattern across age categories (summarized in Figure 3). Namely, younger patients (aged 20–39) only reported moderate and severe ED, whereas older patients also complained of mild and mild‐to‐moderate ED, while also showing higher rates of moderate and severe sexual dysfunction.

At baseline, total IIEF‐EF inversely correlated with age (*ρ* = –0.367, *p* = 0.050) and BMI (*ρ* = –0.489, *p* = 0.008), while showing positive associations with the total AddiQoL score (*ρ* = 0.442, *p* = 0.031) and the Fatigue (*ρ* = 0.512, *p* = 0.011) domain score. Conversely, no relationship was detected between IIEF‐EF total score and sex steroid levels, disease duration, and BSA‐adjusted GC dose.

As shown in Table 3, patients with PAI presented with a higher total IIEF‐EF score compared to the SAI group (*p* = 0.048). This difference was maintained after excluding from the analysis hypogonadal patients (23.4 [7.4] vs. 13.8 [9.9], *p* = 0.020), patients with diabetes mellitus (24.0 [7.45] vs. 14.9 [10.5], *p* = 0.023), or both (24.0 [7.45] vs. 13.8 [9.9], *p* = 0.021), but not after adjusting for age (mean estimated difference 5.9 [−1.8–13.5], *p* = 0.133). Moreover, IIEF‐EF scores were similar between eugonadal and hypogonadal men under replacement therapy (19.6 [9.6] vs. 18.8 [11.5], *p* = 0.792) or between patients with or without diabetes mellitus (24.3 [6.9] vs. 18.5 [6.3], *p* = 0.459).

**FIGURE 4:**
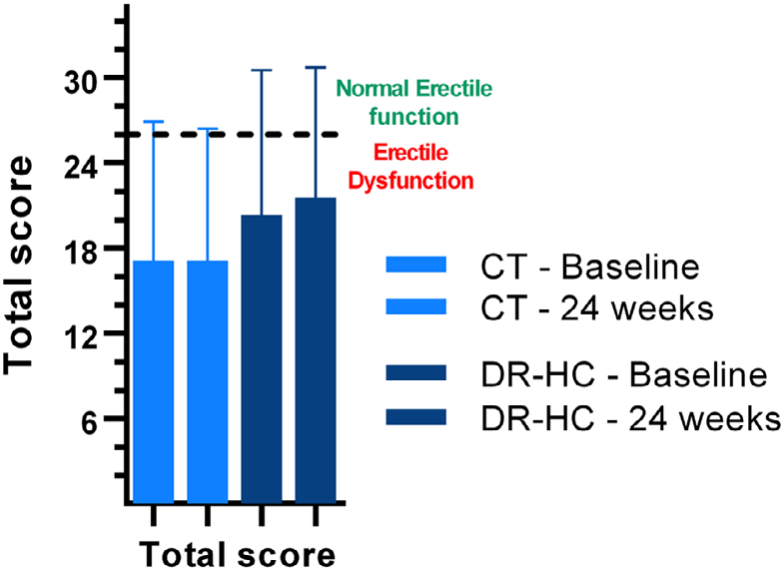
Longitudinal evaluation of International Index of Erectile Function‐Erectile Function (IIEF‐EF) scores. IIEF‐EF total scores of the two treatment groups at baseline and after 24 weeks from randomization. Data are reported as mean (SD). CT = Conventional therapy group, DR‐HC = Dual Release Hydrocortisone group.

No differences between the DR‐HC and CT groups were observed regarding either baseline total IIEF‐EF score (*p* = 0.487) (as shown in Figure 4 and Table 3) or sexual dysfunction rates (*p* = 0.160).

#### 3.2.2 24 weeks

At 24 weeks, 25 men completed the IIEF‐EF questionnaire, and the prevalence of ED was similar to the baseline (60%). The mean IIEF‐EF score was 20.0 (9.3). None of the patients with normal IIEF‐EF scores at baseline developed ED at 24 weeks. Two patients in the CT group showed worse sexual function at 24 weeks (from “mild‐to‐moderate” to “severe” ED and from “mild” to “moderate” ED, respectively), while one patient in the CT group with severe ED at baseline improved to moderate ED at 24 weeks.

No significant associations were observed between the variations from the baseline of IIEF‐EF and AddiQoL scores, both total and in single domains. The comparison between DR‐HC and CT showed no treatment‐related effects in terms of change in ED prevalence and severity.

No differences were found in the variation of IIEF‐EF total score between DR‐HC and CT, even after adjusting for age, BMI, disease duration, baseline IIEF‐EF score, type of AI, and the presence of diabetes mellitus [mean estimated difference: 0.844 (−1.6–3.3); *p* = 0.481)]. In the DR‐HC group, sex steroids showed no significant variations compared to baseline (Table S2), nor were they associated with ED prevalence and severity.

## 4 DISCUSSION

Sexual health is often an underexplored aspect of routine clinical practice, especially in rare diseases.^33^

To our knowledge, this is the first study aiming to assess the prevalence of sexual dysfunction in a population of AI patients under adequate, stable replacement, and to investigate the correlations with steroid hormones, QoL, and replacement regimen.

### 4.1 Female sexual function

The investigation of sexual function in our female patients’ cohort has highlighted several alterations, especially in the post‐menopausal group. Studies on sexually active female patients with AI suggested a normal prevalence of sexual interest.^16^ In our entire cohort (aged 28–77 years), 64% of AI women were sexually active at baseline, in line with normative data from population studies.^34^ However, in our study, only 28.5% of menopausal women were sexually active, a percentage even lower than those observed in population studies,^35^ suggesting a significant reduction in sexual drive after menopause, when adrenal androgens might physiologically exert a more prominent role in female sex steroid homeostasis.

Similar to the results on sexual interest, our analysis revealed lower scores across all domains of the FSFI questionnaire in sexually active post‐menopausal patients compared to pre‐menopausal women.

In sexually active pre‐menopausal women with AI, our results indicated scores compatible with sexual dysfunction in 38.8% of patients, a percentage slightly higher but overall comparable to that reported by a recent meta‐analysis on healthy women (36.3%).^36^

Analyzing our whole female cohort, including sexually inactive women, we observed a significantly diminished sexual desire, indicated by a domain score lower than 5,^37^ in 94% of patients, including all menopausal women, confirming that the desire domain is indeed one of the most affected in women with AI.^21^

### 4.2 Male sexual function

Results from the male population highlighted unexpectedly high rates of sexual dysfunction. Sexual function (in terms of sexual initiative and satisfaction) and ED have been rarely evaluated in AI patients, with only one study showing an increased prevalence of erectile dysfunction in 12 PAI males at diagnosis, that significantly improved within two months of initiating GC and mineralocorticoid replacement therapy.^24^

In our study, we evaluated for the first time patients under stable, long‐term GC replacement, with an impressive rate of ED prevalence (57% of participants), which is notably higher than in the general population.^38^ Moreover, even though the prevalence of ED in healthy men shows great variability among different studies and subgroups,^38^, ^39^, ^40^ age‐stratification showed consistently higher prevalence of ED in our cohort (30% and 74% in patients younger and older than 40 years, respectively) compared to matched healthy individuals.^41^

Additionally, SAI patients exhibited lower IIEF‐EF scores compared to PAI, although this difference did not remain statistically significant after adjusting for age, suggesting the presence of age‐related confounding effects rather than intrinsic etiology‐related differences.

Overall, ED can result from various underlying factors, including psychogenic and organic elements such as hypogonadism, pharmacological interferences, vascular and neurogenic diseases, diabetes, and central obesity.^42^, ^43^, ^44^, ^45^, ^46^ In our cohort, the IIEF‐EF score negatively correlated with BMI, potentially reflecting the well‐known influence of metabolic derangements on erectile function in the general male population.^46^ However, our data collectively suggest a limited impact of organic factors on the pathogenesis of ED in males with AI, since no differences in IIEF‐EF scores were observed between eugonadal and hypogonadal patients receiving testosterone therapy, nor between patients with or without diabetes mellitus. While this might be attributable to several factors, including the relatively small sample size and the optimized control of such comorbidities, our findings might suggest that male sexual dysfunction in AI can be influenced by other disease‐specific mechanisms.

### 4.3 Androgens

The causative factors of sexual dysfunction in men and women with AI have seldom been investigated per se.

In the early 2000s, two randomized placebo‐controlled studies conducted in women with PAI and SAI suggested a potential positive effect of DHEA replacement on sexual interest,^47^, ^48^ frequency of sexual thoughts or fantasies, and mental and physical satisfaction related to sex.^47^ However, these findings were not consistently replicated in subsequent studies,^20^, ^23^, ^49^, ^50^, ^51^ with a meta‐analysis reporting no beneficial effects of DHEA replacement on sexual function in female AI patients^22^ even after long‐term exposure.^22^, ^49^

In line with these reports, our study also revealed no significant correlation between sexual function and androgen levels in our female cohort, even after stratifying for gonadal status. However, it is worth noticing that a trend towards a positive association between testosterone levels and lubrication was observed. This finding aligns with the known beneficial effects of testosterone therapy in women affected by hypoactive sexual desire disorder,^52^ as highlighted by the current guidelines on androgen therapy in women and the global Consensus Position Statement on the use of testosterone therapy for women.^53^, ^54^

Similarly, we observed no correlation between adrenal androgens and sexual dysfunction in our male cohort, consistent with previous clinical trials^49^, ^50^ that found no significant improvement in sexual function with DHEA replacement therapy in male patients with primary or secondary AI.

Collectively, our data support that adrenal androgen deficiency seems to exert little to no effect on sexual function in AI patients. Indeed, in males and premenopausal women with AI, androgen deficiency is generally compensated by gonadal production,^51^, ^55^ and there is no established lower threshold of circulating androgens that can be used to identify patients, particularly women, with impaired sexual function. It is possible, then, that postmenopausal women could be a more suitable population for exploring the effects of androgen replacement, given the increased impairment in sexual function that we observed in this subgroup compared to the general population and premenopausal AI women.

### 4.4 Quality of life

The most striking result of our trial is the strong association between QoL, as assessed by the disease‐specific AddiQoL questionnaire, and sexual function in both male and female patients with AI. Specifically, the total score of AddiQoL and the fatigue domain were positively correlated with both the FSFI and IIEF‐EF total scores, underscoring a close relationship between health‐related QoL, energy levels, and sexuality in individuals with AI. This observation would appear consistent with the study from Arnaldi and colleagues conducted on newly diagnosed PAI men, in which sexual dysfunction was more pronounced at the time of diagnosis but showed improvement after two months of treatment initiation.^24^ The authors speculated that symptoms experienced at diagnosis (i.e., fatigue, hypotension, and nausea) adversely affected sexual function. Consequently, it is plausible to suggest that patients with fewer AI‐related symptoms may also enjoy better sexual health.

### 4.5 Effects of DR‐HC

Lastly, our study aimed to investigate the potential impact of a more physiological GC regimen on sexual function. GCs exert a wide spectrum of activities that extend to the regulation of cognition and behavioral function,^56^ and impaired cortisol secretion, as observed in AI, can lead to several systemic alterations that detrimentally affect QoL.^1^ Hence, it is plausible that in patients with AI, a non‐physiological cortisol replacement may also influence sexual function. Recently, DR‐HC, a once‐daily GC formulation that better mimics the circadian rhythm of cortisol secretion, has been associated with improved metabolic and immune outcomes along with overall QoL in patients enrolled in the DREAM trial.^8^ In our cohort, female patients treated with DR‐HC showed a positive association between the increase in quality‐of‐life questionnaire scores and the change in sexual function scores.

Nevertheless, there was no significant change in sexual function after 24 weeks of DR‐HC treatment compared to the CT group in both the male and female populations. Consistently with our own findings, the only study conducted thus far reporting data on DR‐HC and sexual function in PAI women found no significant differences between patients treated with DR‐HC and those receiving conventional formulations.^21^ however, it is also possible that the indirect effects of therapy switch on sexual function could be unmasked by a more prolonged observation, as previously suggested,^48^ thanks to the positive effects of DR‐HC on metabolic outcomes and inflammation, as described by the main trial.^8^

According to our results on the impact of perceived well‐being on sexual function, a more physiological replacement regimen may also indirectly enhance sexual function through the amelioration of overall QoL. Additional studies with larger cohorts and longer follow‐up periods are necessary to validate and further explore these findings.

### 4.6 Strengths and limitations

This study possessed several strengths, including its prospective, randomized controlled design and the use of gold‐standard methodologies to assess sex steroid levels. However, some limitations should be acknowledged. Firstly, the assessment of sexual function via questionnaires was not compared against a healthy, age‐matched control group, even though normative cut‐offs of both male and female questionnaires have been published.^57^ Secondly, 29% of patients originally enrolled in the DREAM trial did not consent to sexual function evaluation. These patients were older than the included cohort, which might represent a potential selection bias. However, this may also be considered a strength of our study, as younger patients, more open to discussing their experiences, may offer a more accurate reflection of a sexual dysfunction directly attributable to the disease, rather than age‐related factors. Moreover, the two groups did not significantly differ in the remaining clinical and demographic characteristics, suggesting that the analyzed sample might accurately reflect the original cohort. Furthermore, questionnaire evaluation may not be sufficient to adequately reflect the complexity of sexual dysfunction, which is a multifactorial condition requiring an integrated approach; additionally, self‐reporting via questionnaires may be subject to bias due to altered self‐perception, as indicated by the discrepancy between sexual function results reported by AI patients and their partners in previous studies.^48^ Moreover, we did not assess orgasmic function and intensity with dedicated tools.^58^, ^59^ However, this research is currently ongoing in our clinic both in basal and in treated male and female patients with AI. Lastly, the hormonal evaluation did not include active metabolites, even though the aim of our study was to assess the potential effects of adrenal androgens on our study outcomes, rather than evaluate the relationship between sex steroids and sexual function.

## 5 CONCLUSIONS

Our study has provided insight into the prevalence and causes of sexual dysfunction among people living with adrenal insufficiency, showing a markedly increased incidence of erectile dysfunction in men across all ages and reduced desire in 94% of women. Importantly, we found no direct correlation between adrenal androgens and sexual health outcomes, and a largely neutral impact of short‐term dual‐release hydrocortisone on sexual well‐being, even though a more prolonged exposure might have an indirect effect by improving metabolic outcomes and well‐being. Our findings also shed light on the significant role played by reduced quality of life, commonly observed in patients with adrenal insufficiency, in influencing sexual function. All in all, our results suggest that sexual dysfunction in adrenal insufficiency is a largely under‐investigated and under‐addressed topic, especially in the male and post‐menopausal population, and should not be merely considered as a consequence of adrenal androgen deficiency, but rather as a multifactorial condition that requires specific counseling and targeted therapeutic interventions.

## AUTHOR CONTRIBUTIONS

Andrea M. Isidori and Mary Anna Venneri contributed to the study's conception and design. Valeria Hasenmajer, Dario De Alcubierre, Francesca Sciarra, and Davide Ferrari contributed to material preparation and data collection. Valeria Hasenmajer and Dario De Alcubierre performed statistical analysis. The first draft of the manuscript was written by Valeria Hasenmajer and Dario De Alcubierre. All the other authors reviewed, edited, and commented on previous versions of the manuscript. All authors read and approved the final manuscript.

## CONFLICT OF INTEREST STATEMENT

The authors declare no conflict of interest.

## Supporting information

Supporting Information

Supporting Information

## Data Availability

The dataset generated and analyzed during the current study is not publicly available but is available from the corresponding author at a reasonable request.
