# Supplementary material for: Exploring sexual function in adrenal insufficiency: findings from the Dual RElease hydrocortisone versus conventionAl glucocorticoid replaceMent therapy in hypocortisolism (DREAM) trial
Source: Andrology. 2024 Mar 28;13(2):302–13. doi: 10.1111/andr.13635 (PMC11815544; doi:10.1111/andr.13635)
Supplement: Supplementary file 1 — Supporting Information [file ANDR-13-302-s001.docx]

**Supplementary Table 1: Change in sex steroids between baseline and follow-up and treatment-related differences in female AI patients.**

|  | **CT** | | **DRHC** | | **Treatment-related difference** **^†^** | ***p^b^*** |
| --- | --- | --- | --- | --- | --- | --- |
|  | Change at 24 weeks | ***p*^a^** | Change at 24 weeks | ***p*^a^** |  |  |
| Total Testosterone (ng/mL) | 0.02 (-0.05 to 0.08) | .735 | 0.04 (-0.01 to 0.09) | .109 | 0.05 (-0.03 to 0.12) | .204 |
| Estradiol (pg/mL) | 4.2 (-7.5 to 16.0) | .317 | 1.0 (-23.0 to 25.0) | .913 | -6.0 (-27.0 to 16.0) | .536 |
| Androstenedione (ng/mL) | 0.93 (-1.73 to 3.59) | .310 | -0.27 (-1.94 to 1.40) | .697 | -1.54 (-4.53 to 1.45) | .284 |
| DHEA-S (ng/mL) | -11.3 (-50.8 to 28.2) | .345 | -50.1 (-226.9 to 126.7) | .347 | 14.3 (-30.6 to 59.3) | .476 |
| 17-OH-Progesterone (ng/mL) | 0.11 (-0.26 to 0.47) | .463 | -0.30 (-0.81 to 0.20) | .122 | -0.52 (-1.38 to 0.35) | .206 |

Evaluation of sex steroids change at 24 weeks in female AI patients. Data are reported as mean change (95%CI) from baseline. p^a^ refers to the change within the single CT or DR-HC group compared to baseline, while p^b^ refers to differences in sex steroids change between CT and DRHC after correction for age and baseline outcome. ^†^Adjusted for age and baseline outcome.

CT = Conventional therapy, DR-HC= Dual-Release Hydrocortisone
