# Supplementary material for: Exploring sexual function in adrenal insufficiency: findings from the Dual RElease hydrocortisone versus conventionAl glucocorticoid replaceMent therapy in hypocortisolism (DREAM) trial
Source: Andrology. 2024 Mar 28;13(2):302–13. doi: 10.1111/andr.13635 (PMC11815544; doi:10.1111/andr.13635)
Supplement: Supplementary file 2 — Supporting Information [file ANDR-13-302-s002.docx]

**Supplementary Table 2: Change in sex steroids between baseline and follow-up and treatment-related differences in male AI patients.**

|  | **CT** | | **DRHC** | | **Treatment-related difference^†^** | ***p^b^*** |
| --- | --- | --- | --- | --- | --- | --- |
|  | Change at 24 weeks | ***p^a^*** | Change at 24 weeks | ***p^a^*** |  |  |
| Total Testosterone (ng/mL) | 0.14 (-0.31 to 0.59) | .391 | -0.54 (-1.50 to 0.43) | .180 | -0.21 (-1.30 to 0.88) | .582 |
| Estradiol (pg/mL) | -7.5 (-67.6 to 52.6) | .655 | -9.0 (-23.4 to 5.4) | .144 | -14.0 (-39.0 to 10.0) | .188 |
| Androstenedione (ng/mL) | 0.24 (-0.53 to 1.02) | .317 | 0.14 (-0.29 to 0.56) | .420 | -0.16 (-1.06 to 0.75) | .700 |
| DHEA-S (ng/mL) | 42.2 (-92.1 to 176.6) | .391 | -17.9 (-96.3 to 60.5) | .273 | -63.7 (-205.1 to 77.7 | .279 |
| 17-OH-Progesterone (ng/mL) | 0.08 (-0.28 to 0.43) | .547 | 0.74 (-0.20 to 1.68) | 138 | 0.38 (-0.27 to 1.04) | .195 |

Evaluation of sex steroids change at 24 weeks in male AI patients. Data are reported as mean change (95%CI) from baseline. p^a^ refers to the change within the single CT or DR-HC group compared to baseline, while p^b^ refers to differences in sex steroids change between CT and DRHC after correction for age and baseline outcome. ^†^Adjusted for age and baseline outcome.

CT = Conventional therapy, DR-HC= Dual-Release Hydrocortisone
